# Supplementary material for: Multivalency drives interactions of alpha-synuclein fibrils with tau
Source: PLoS One. 2024 Sep 10;19(9):e0309416. doi: 10.1371/journal.pone.0309416 (PMC11386428; doi:10.1371/journal.pone.0309416)
Supplement: S1 Fig — Absorbance spectra a) of acetylated αS and acetylated pS129 αS used to calculated concentrations of both proteins prior to MALDI-TOF (ε@280 nm = 5960 M-1cm-1). Both proteins were mixed in equimolar amounts and spotted for b) MADLI-TOF analysis. The relatively equal spectral peak heights of the MALDI-TOF spectra and the expected mass shift indicate that most, if not all, the αS is phosphorylated after the co-expression of NatB and PLK2 plasmids (see Materials and Methods for details). (PDF) [file pone.0309416.s001.pdf]

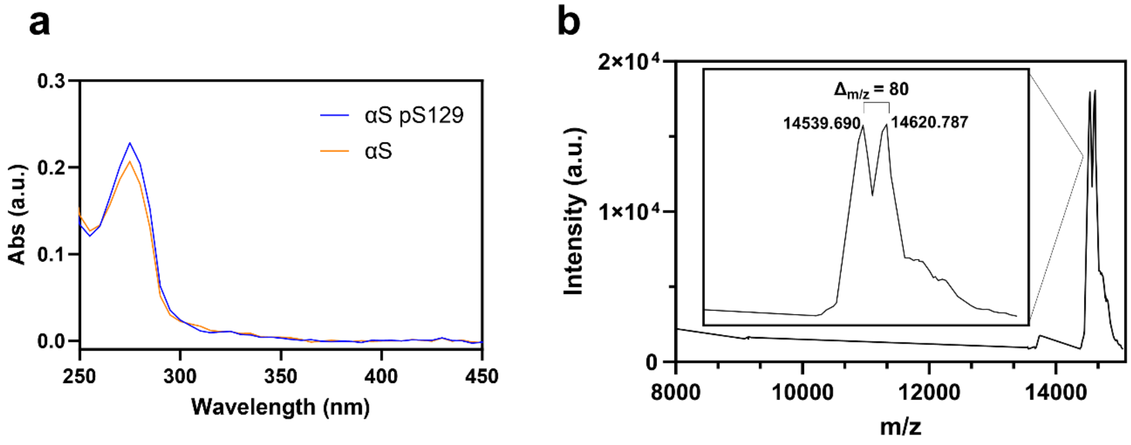

S1 Fig. Absorption and MALDI-TOF spectra of αS pS129. Absorbance spectra a) of acetylated αS and acetylated pS129 αS used to calculated concentrations of both proteins prior to MALDI-TOF (ε@280 nm = 5960 $M^{-1}cm^{-1}$). Both proteins were mixed in equimolar amounts and spotted for b) MADLI-TOF analysis. The relatively equal spectral peak heights of the MALDI-TOF spectra and the expected mass shift indicate that most, if not all, the αS is phosphorylated after the co-expression of NatB and PLK2 plasmids (see Materials and Methods for details).
