# Supplementary material for: Multivalency drives interactions of alpha-synuclein fibrils with tau
Source: PLoS One. 2024 Sep 10;19(9):e0309416. doi: 10.1371/journal.pone.0309416 (PMC11386428; doi:10.1371/journal.pone.0309416)
Supplement: S2 Fig — a) Fibrils of full length αS and αS1-100 was imaged by transmission electron microscopy (TEM) as described in the Material and Methods to ensure subsequent seeds formed from fibrillar samples. b) Fibrils of full length αS imaged by TEM post sonication as described in Material and Methods. c) SDS-PAGE gel showing the amount of protein present in the supernatant (S) and pellet (P) of full-length αS versus αS1-100 fibrils and ‘seed’ full-length αS versus αS1-100 post-sonication as described in Materials and Methods. d) Quantification by ImageJ of average amount of protein based on both gels in the pellet versus the pellet and the supernatant summed shows most of the protein for full-length is in the pellet while for αS1-100, 18% remains as soluble species. For the seeds, 75% of full-length αS remains as soluble species while 33% remains for αS1-100. (PDF) [file pone.0309416.s002.pdf]

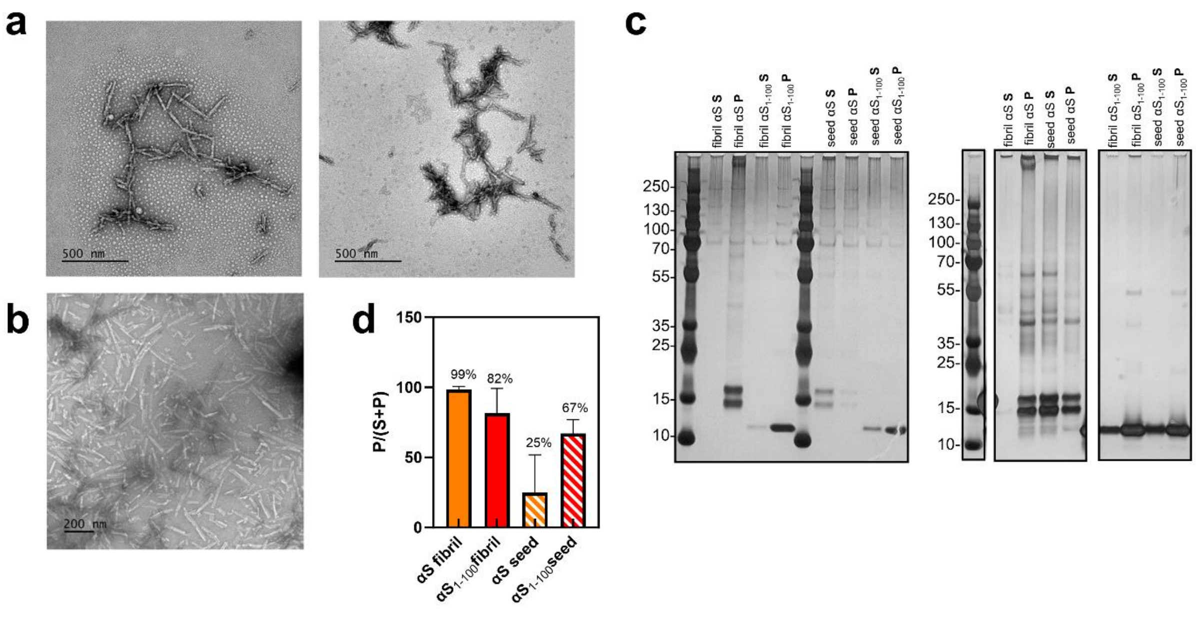

S2 Fig. Characterization of αS seeds. a) Fibrils of full length αS and αS_{1-100} was imaged by transmission electron microscopy (TEM) as described in the Material and Methods to ensure subsequent seeds formed from fibrillar samples. b) Fibrils of full length αS imaged by TEM post sonication as described in Material and Methods. c) SDS-PAGE gel showing the amount of protein present in the supernatant (S) and pellet (P) of full-length αS versus αS_{1-100} fibrils and 'seed' full-length αS versus αS_{1-100} post-sonication as described in Materials and Methods. c) Quantification by ImageJ of average amount of protein based on both gels in the pellet versus the pellet and the supernatant summed shows most of the protein for full-length is in the pellet while for αS_{1-100}, 18% remains as soluble species. For the seeds, 75% of full-length αS remains as soluble species while 33% remains for αS_{1-100}.
