# Supplementary material for: Multivalency drives interactions of alpha-synuclein fibrils with tau
Source: PLoS One. 2024 Sep 10;19(9):e0309416. doi: 10.1371/journal.pone.0309416 (PMC11386428; doi:10.1371/journal.pone.0309416)
Supplement: S3 Fig — a) For tauPRR, comparison of three fit equations: (1) one diffusing component (protein only); (2) one diffusing component with exponential decay (triplet); (3) two diffusing components (protein and free dye). For all fits, only s was fixed, and all other parameters were allowed to float. b) The equation using the triplet state with the autocorrelation function G(τ) was calculated as a function of the delay time τ where G(τ) is the autocorrelation function, N is the number of molecules in the focal volume, A is the fraction of the exponential contribution, τT is the triplet lifetime, τD is the translational diffusion time, s is the ratio of radial to axial dimensions of the focal volume. The other fit equations are found in the main manuscript. Fit parameters for each model are reported. c) Considering the increased number of free parameters in both the triplet and two component diffusion fits, the fit quality was not improved over the one component fit, as displayed by an F-test showing insufficient evidence to support a difference in the variances of the residuals. (PDF) [file pone.0309416.s003.pdf]

a

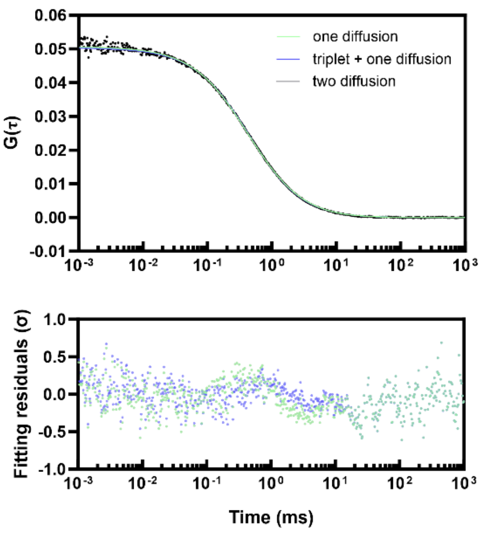

b

$$G(\tau) = \frac{1}{N}\left(\frac{1}{1-A} \times \left(1 - A + Ae^{\frac{-\tau}{\tau_T}}\right) \times \frac{1}{1+\frac{\tau}{\tau_D}} \times \sqrt{\frac{1}{1+\frac{s^2\,\tau}{\tau_D}}}\right)$$

N	τ_D
20	0.43

N	τ_D	τ_T	A
22	0.46	1.2	0.092

N	τ_{D1}	τ_{D2}	A	Q
17	0.43	0.43	0.57	0.67

c

$$Fstat = \frac{S_y^2}{S_x^2}$$

$$s_y^2 = \frac{\Sigma(y-\bar{y})}{n-1}$$

$$s_x^2 = \frac{\Sigma(x-\bar{x})}{n-1}$$

Fstat for single versus triplet = 1.0007263

Fstat for single versus two comp = 1.000001

Fcritical = 1.139

Fstat < Fcritical, equal variances; cannot reject null hypothesis

S3 Fig. Comparison of FCS fitting models. a) For tauPRR, comparison of three fit equations: (1) single diffusing component (protein only); (2) single diffusing component with exponential decay (triplet); (3) two diffusing components (protein and free dye). For all fits, only s was fixed, and all other parameters were allowed to float. b) The equation using the triplet state with the autocorrelation function G(τ) was calculated as a function of the delay time τ where G(τ) is the autocorrelation function, N is the number of molecules in the focal volume, A is the fraction of free tau, τT is the tau triplet, τD is the translational diffusion time, s is the ratio of radial to axial dimensions of the focal volume. The other fit equations are found in the main manuscript. Fit parameters for each model are reported. c) Considering the increased number of free parameters in both the triplet and two component diffusion fits, the fit quality was not improved over the one component fit, as displayed by an F-test showing insufficient evidence to support a difference in the variances of the residuals.
