# Supplementary material for: Multivalency drives interactions of alpha-synuclein fibrils with tau
Source: PLoS One. 2024 Sep 10;19(9):e0309416. doi: 10.1371/journal.pone.0309416 (PMC11386428; doi:10.1371/journal.pone.0309416)
Supplement: S4 Fig — Aberrant curves disproportionally weigh the averaged autocorrelation at higher αS concentrations. Representative individual autocorrelation curves using labeled tau4R in the absence (a) or presence of αS monomer (b), full-length seeds (c) or truncated seeds (d). The correlation curves are gray and the fit to the appropriate diffusion equation as described in the Materials and Methods are in red. The plots in the left-hand column display all the collected autocorrelation curves; the plots in the right-hand column display the remaining autocorrelation curves following the SSR analysis described in the Materials and Methods. Very few of the curves (as quantified in tables below for each construct) fall outside the main distribution, so that discarding aberrant curves with extremely high τD values does not change interpretation of the data. (PDF) [file pone.0309416.s004.pdf]

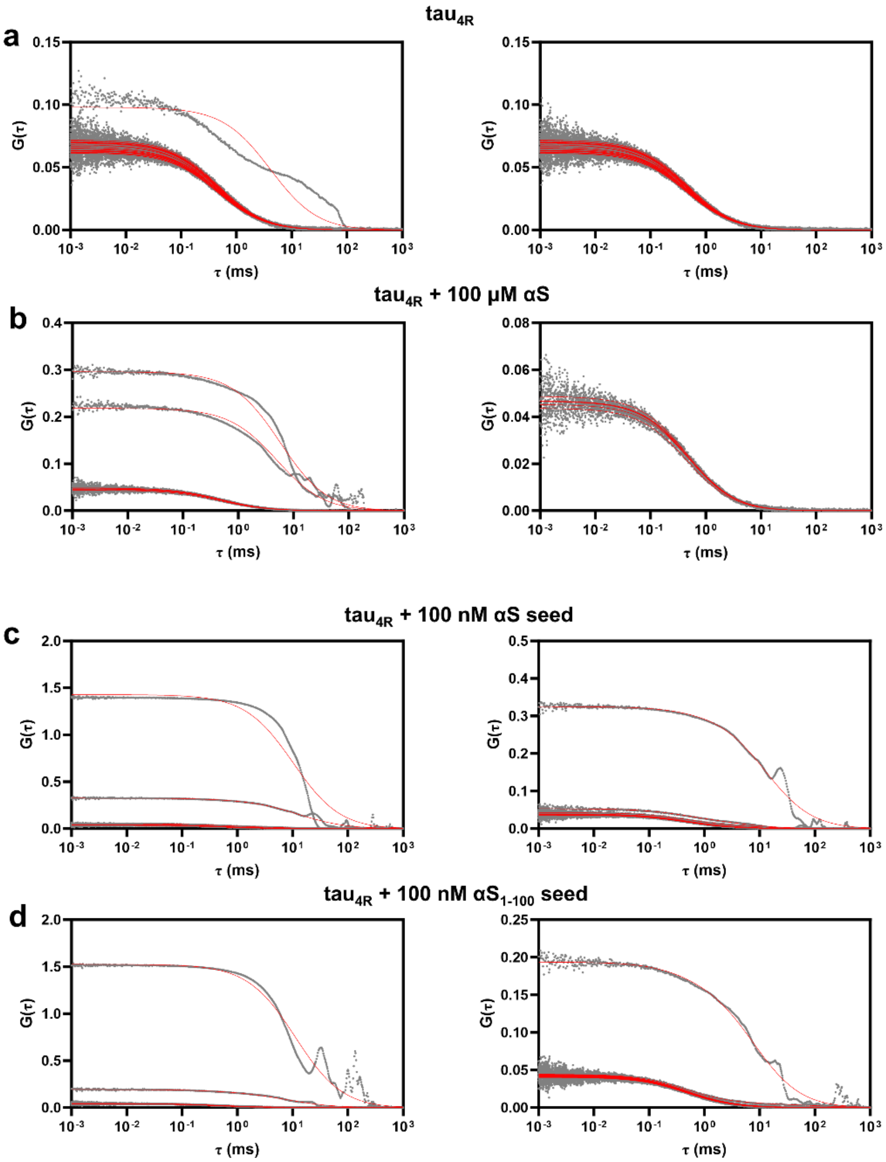


**S4 Fig. Representative individual autocorrelation curves with monomeric and seed αS.** Aberrant curves disproportionally weigh the averaged autocorrelation at higher αS concentrations. Representative individual autocorrelation curves using labeled tau4R in the absence (a) or presence of αS monomer (b), full-length seeds (c) or truncated seeds (d). The correlation curves are gray and the fit to the appropriate diffusion equation as described in the Materials and Methods are in red. The plots in the left-hand column display all the collected autocorrelation curves; the plots in the right-hand column display the remaining autocorrelation

curves following the SRR analysis described in the Materials and Methods. Very few of the curves (as quantified in tables below for each construct) fall outside the main distribution, so that discarding aberrant curves with extremely high $\tau_D$ values does not change interpretation of the data.
