# Supplementary material for: Multivalency drives interactions of alpha-synuclein fibrils with tau
Source: PLoS One. 2024 Sep 10;19(9):e0309416. doi: 10.1371/journal.pone.0309416 (PMC11386428; doi:10.1371/journal.pone.0309416)
Supplement: S5 Fig — Tau binds to αS monomer in a weakly concentration dependent manner. Unlabeled αS was added to fluorescently labeled tau as described in the Materials & Methods. Normalized τD for a) tau4R and b) tauPRR. (PDF) [file pone.0309416.s005.pdf]

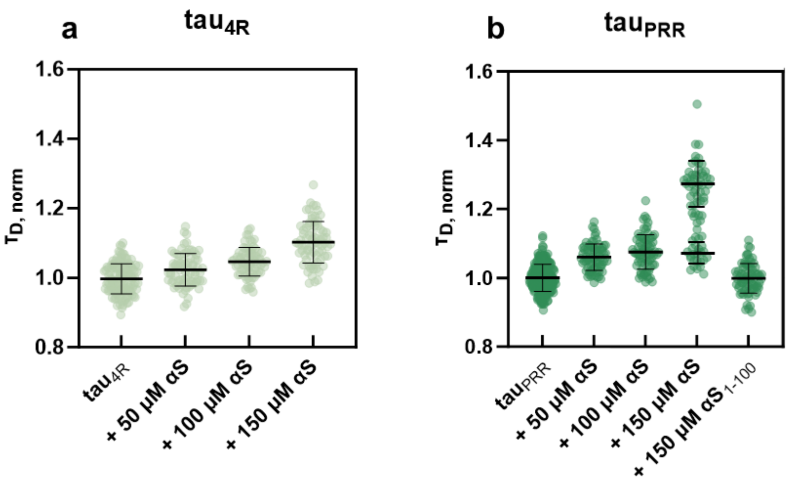

S5 Fig. τ_D for monomer tau_{4R} and tau_{PRR} at increasing concentrations. Tau binds to αS monomer in a weakly concentration dependent manner. Unlabeled αS was added to fluorescently labeled tau as described in the Materials & Methods. Normalized τ_D for a) tau_{4R} and b) tau_{PRR}.
