# Supplementary material for: Multivalency drives interactions of alpha-synuclein fibrils with tau
Source: PLoS One. 2024 Sep 10;19(9):e0309416. doi: 10.1371/journal.pone.0309416 (PMC11386428; doi:10.1371/journal.pone.0309416)
Supplement: S6 Fig — eGFP interacts weakly with αS monomer. Plots shown τD, norm for a) eGFP and b) eGFP with 150 μM αS. Mean τD, norm and SD calculated for a minimum of three measurements with αS. % diff = [τD(+αS)-τD(-αS)]/τD(-αS). (PDF) [file pone.0309416.s006.pdf]

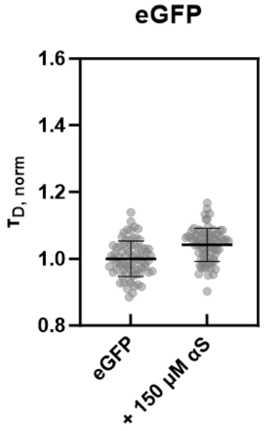

construct	$\tau_{D,\ norm}$ - αS	$\tau_{D,\ norm}$ + αS	% diff + αS
eGFP	1.00±0.053	1.04±0.050	4

S6 Fig. Interaction of eGFP with monomer αS. eGFP interacts weakly with αS monomer. Plots shown $\tau_{D,\ norm}$ for a) eGFP and b) eGFP with 150 µM αS. Mean $\tau_{D,\ norm}$ and SD calculated for a minimum of three measurements with αS. % diff = $[\tau_D(+\alpha S)-\tau_D(-\alpha S)]/\tau_D(-\alpha S)$.
