# Supplementary material for: Multivalency drives interactions of alpha-synuclein fibrils with tau
Source: PLoS One. 2024 Sep 10;19(9):e0309416. doi: 10.1371/journal.pone.0309416 (PMC11386428; doi:10.1371/journal.pone.0309416)
Supplement: S7 Fig — Representative distributions of τD values for a) tauPRR and tauPRR plus b) αS monomer or c) seed (τD2app). Both a) and b) show symmetric distributions, while c) is asymmetric with a tail of higher τD2app values. (PDF) [file pone.0309416.s007.pdf]

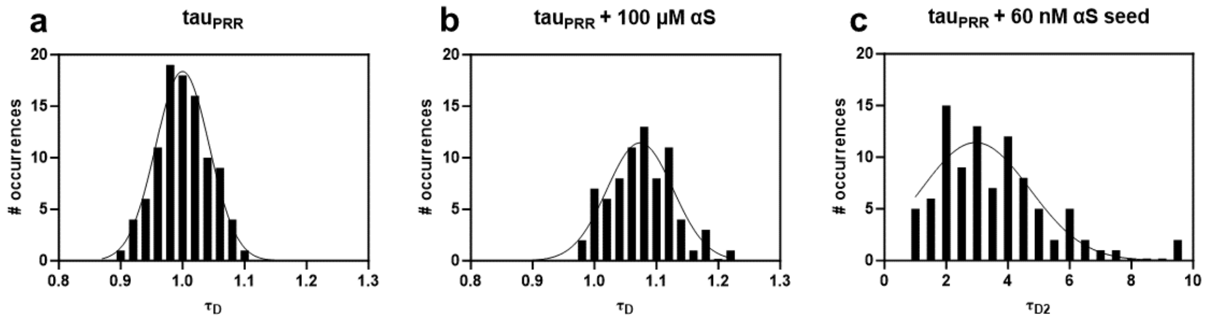

S7 Fig. Representative distributions of τ_D values. Representative distributions of τ_D values for a) tau$_{PRR}$ and tau$_{PRR}$ plus b) αS monomer or c) seed (τ_{D2app}). Both a) and b) show symmetric distributions, while c) is asymmetric with a tail of higher τ_{D2app} values.
