# Supplementary material for: Multivalency drives interactions of alpha-synuclein fibrils with tau
Source: PLoS One. 2024 Sep 10;19(9):e0309416. doi: 10.1371/journal.pone.0309416 (PMC11386428; doi:10.1371/journal.pone.0309416)
Supplement: S8 Fig — Post-aggregation samples for tau1N4R (first panel), followed by samples with αS and αS1-100 seeds, middle and right panel respectively, were imaged on a T12 Tencai microscope. (PDF) [file pone.0309416.s008.pdf]

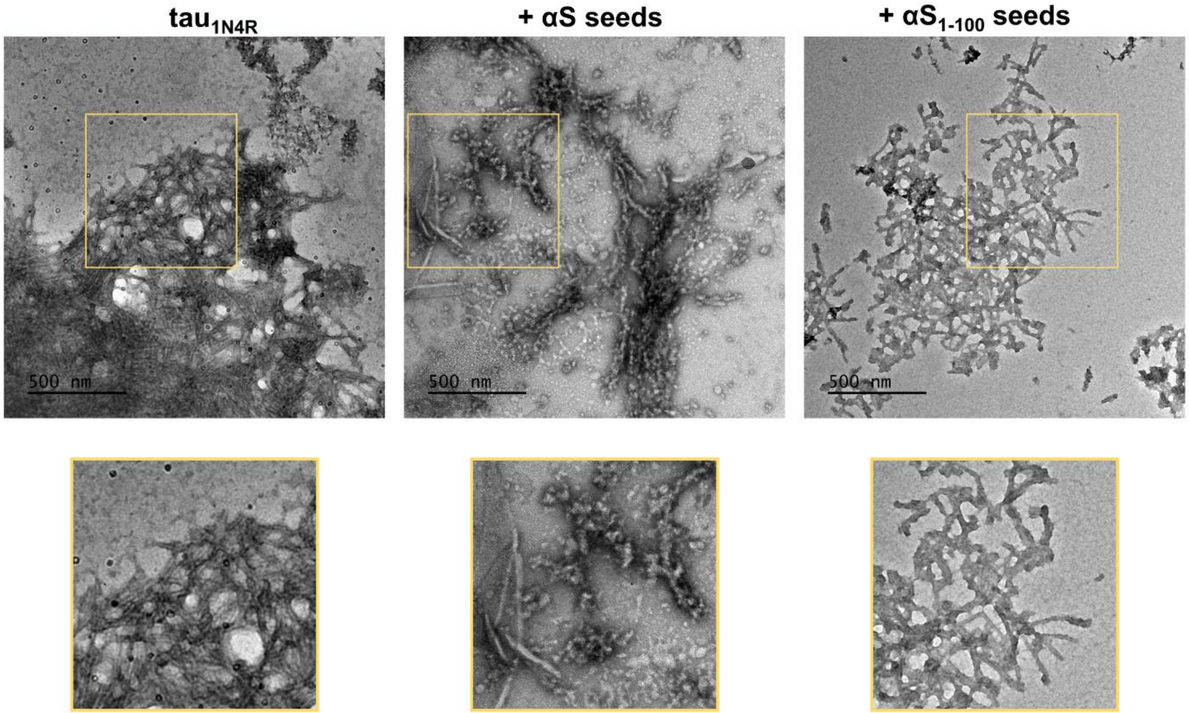

S8 Fig. Tau aggregates are fibrillar. Post-aggregation samples for tau_{1N4R} (first panel), followed by samples with αS and $αS_{1\text{-}100}$ seeds, middle and right panel respectively, were imaged on a T12 Tencai microscope.
