# Supplementary material for: Multivalency drives interactions of alpha-synuclein fibrils with tau
Source: PLoS One. 2024 Sep 10;19(9):e0309416. doi: 10.1371/journal.pone.0309416 (PMC11386428; doi:10.1371/journal.pone.0309416)
Supplement: S9 Fig — Charge plots generated using CIDER outline the distribution of positive versus negative charges in the PRR of tau compared to the repeats, as well as the distribution in the domains of αS. The numbering for tau is for the longest tau isoform, 2N4R; 1N4R lacks one of the N-terminal inserts (indicated in pink on the plot). CIDER analysis for tau and αS constructs are listed in the table, NCPR (net charge per residue), κ (kappa) charge patterning parameter, f+ (fraction of positive residues), and f- (fraction of negative residues). (PDF) [file pone.0309416.s009.pdf]

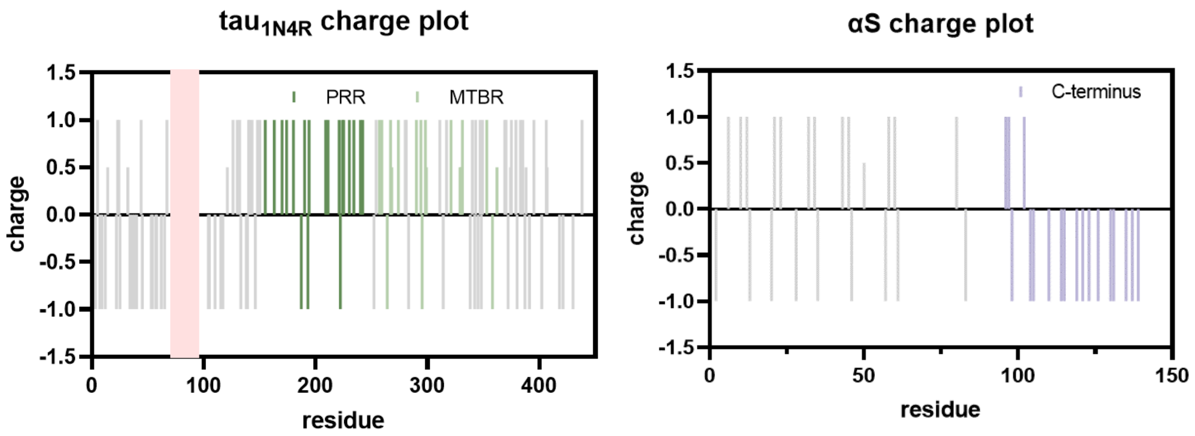

construct	length	NCPR	κ	f+	f-
tau_{1N4R}	414	0.014	0.181	0.138	0.123
$tau_{N\text{-}terminus}$	119	-0.143	0.211	0.101	0.244
tau_{4R}	133	0.075	0.107	0.158	0.083
tau_{PRR}	98	0.143	0.111	0.173	0.030
αS	140	-0.064	0.172	0.107	0.171
$αS_{1\text{-}100}$	100	0.040	0.082	0.140	0.100
$αS_{101\text{-}140}$	40	-0.325	0.091	0.025	0.350

S9 Fig. Charge plots of αS and tau generated using CIDER. Charge plots generated using CIDER outline the distribution of positive versus negative charges in the PRR of tau compared to the repeats, as well as the distribution in the domains of αS. The numbering for tau is for the longest tau isoform, 2N4R; 1N4R lacks one of the N-terminal inserts (indicated in pink on the plot). CIDER analysis for tau and αS constructs are listed in the table, NCPR (net charge per residue), κ (kappa) charge patterning parameter, f+ (fraction of positive residues), and f- (fraction of negative residues).
