# Supplementary material for: Multivalency drives interactions of alpha-synuclein fibrils with tau
Source: PLoS One. 2024 Sep 10;19(9):e0309416. doi: 10.1371/journal.pone.0309416 (PMC11386428; doi:10.1371/journal.pone.0309416)
Supplement: S10 Fig — Phosphorylation of monomer αS at pS129 does not enhance interactions with tauPRR. Unlabeled αS was added at concentrations indicated on plot. Both αS and αSpS129 show mild, concentration-dependent increases in binding, reflecting weak interactions. Mean τD, norm and SD calculated for a minimum of three measurements with increasing concentration of αS. % diff = [τD(+αS)-τD(-αS)]/τD(-αS). (PDF) [file pone.0309416.s010.pdf]

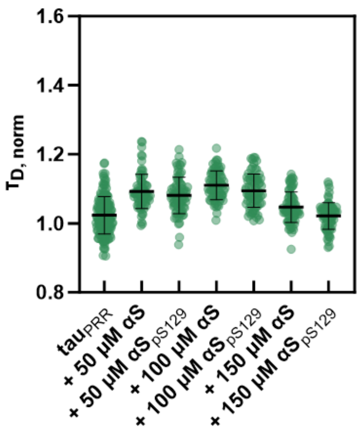


| construct | $\tau_{D,\ norm}$ | % diff |
|---|---|---|
| $tau_{PRR}$ | 1.00±0.039 | - |
| + 50 μM αS | 1.03±0.047 | 3 |
| + 100 μM αS | 1.05±0.039 | 5 |
| + 150 μM αS | 1.05±0.042 | 5 |
| + 50 μM $\alpha S_{pS129}$ | 1.02±0.050 | 2 |
| + 100 μM $\alpha S_{pS129}$ | 1.04±0.045 | 4 |
| + 150 μM $\alpha S_{pS129}$ | 1.03±0.036 | 4 |

**S10 Fig. $\tau_D$ with monomer αS pS129.** Phosphorylation of monomer αS at pS129 does not enhance interactions with $tau_{PRR}$. Unlabeled αS was added at concentrations indicated on plot. Both αS and $\alpha S_{pS129}$ show mild, concentration-dependent increases in binding, reflecting weak interactions. Mean $\tau_{D,\ norm}$ and SD calculated for a minimum of three measurements with increasing concentration of αS. % diff= $[\tau_D(+\alpha S)-\tau_D(-\alpha S)]/\tau_D(-\alpha S)$.
