# Supplementary material for: Multivalency drives interactions of alpha-synuclein fibrils with tau
Source: PLoS One. 2024 Sep 10;19(9):e0309416. doi: 10.1371/journal.pone.0309416 (PMC11386428; doi:10.1371/journal.pone.0309416)
Supplement: S1 Table — Table summarizing autocorrelation data. Analysis of initial data sets (column 1) by the SSR as described in the Materials and Methods resulted discarding a few autocorrelation curves (column 2) for each set of measurements. The normalized SSR (column 3) and % curves analyzed (column 4) are also included. (PDF) [file pone.0309416.s011.pdf]

Tau construct (+ monomer αS)	# of curves collected	# curves analyzed	SSR/# curves	% curves analyzed
**tau$_{1N4R}$**	75	73	6.31E-05	97.3
+ 150 μM αS	75	75	6.77E-05	100
**tau$_{4R}$**	125	125	2.76E-05	100
+ 150 μM αS	75	75	2.46E-04	100
+ 100 μM αS	75	72	4.49E-05	96
+ 50 μM αS	75	75	5.93E-05	100
**tau$_{PRR}$**	225	221	1.90E-05	98.2
+ 150 μM αS$_{1-100}$	75	75	3.81E-05	100
+ 150 μM αS	75	75	4.34E-05	100
+ 100 μM αS	75	75	2.09E-04	100
+ 50 μM αS	75	75	4.65E-05	100
**eGFP**	75	75	1.62E-04	100
+ 150 μM αS	75	75	1.33E-04	100

Tau construct (+ seed αS)	# of curves collected	# curves analyzed	SSR/# curves	% curves analyzed
**tau$_{1N4R}$**	125	123	3.18E-05	98.4
+ 20 nM αS	75	73	1.52E-04	97.3
+ 60 nM αS	75	73	9.30E-05	97.3
+ 100 nM αS	75	71	2.12E-04	94.7
+ 20 nM αS$_{1-100}$	75	75	2.73E-04	100
+ 60 nM αS$_{1-100}$	75	72	6.97E-05	96
+ 100 nM αS$_{1-100}$	75	68	6.71E-04	90.7
**tau$_{4R}$**	150	149	2.93E-05	99.3
+ 20 nM αS	75	73	6.73E-05	97.3
+ 60 nM αS	100	95	3.36E-05	95
+ 100 nM αS	75	71	4.61E-05	94.7
+ 20 nM αS$_{1-100}$	75	73	8.30E-05	97.3
+ 60 nM αS$_{1-100}$	100	93	3.19E-05	93
+ 100 nM αS$_{1-100}$	75	72	5.60E-05	96
**tau$_{PRR}$**	200	199	2.27E-05	99.5
+ 20 nM αS	75	74	6.86E-05	98.7
+ 60 nM αS	100	94	8.81E-05	94
+ 100 nM αS	75	68	6.46E-05	90.7
+ 20 nM αS$_{1-100}$	75	75	1.27E-04	100
+ 60 nM αS$_{1-100}$	100	94	5.90E-05	94
+ 100 nM αS$_{1-100}$	75	72	8.58E-05	96
**tau$_{PRR}$** (5 mM NaCl)	75	74	6.28E-05	98.7
+ 60 nM αS	75	73	2.77E-04	97.3
**tau$_{PRR}$** (50 mM NaCl)	75	75	5.13E-05	100
+ 60 nM αS	75	73	6.12E-05	97.3
**tau$_{PRR}$** (500 mM NaCl)	75	75	1.00E-05	100
+ 60 nM αS	75	74	1.22E-04	98.7
**eGFP**	75	75	1.20E-04	100
+ 60 nM αS	75	75	1.39E-04	100

S1 Table. Analysis of autocorrelation curves discarded through poor fit. Table summarizing autocorrelation data. Analysis of initial data sets (column 1) by the SSR as described in the Materials and Methods resulted discarding a few autocorrelation curves (column 2) for each set of measurements. The normalized SSR (column 3) and % curves analyzed (column 4) are also included.
