# Supplementary material for: Multivalency drives interactions of alpha-synuclein fibrils with tau
Source: PLoS One. 2024 Sep 10;19(9):e0309416. doi: 10.1371/journal.pone.0309416 (PMC11386428; doi:10.1371/journal.pone.0309416)
Supplement: S2 Table — Results of one-tailed t-tests (GraphPad Prism) for tau binding to monomer αS, ns = not significant, t = t value and df degrees of freedom with a Geisser-Greenhouse correction. (PDF) [file pone.0309416.s012.pdf]

tau_{1N4R}	P value	P value significance	t, df
+ 150 µM αS	<0.0001	****	t=8.588, df=140.2
**tau_{4R}**	**P value**	**P value significance**	**t, df**
+ 50 µM αS	<0.0001	****	t=3.940, df=147.2
+ 100 µM αS	<0.0001	****	t=7.956, df=156.2
+ 150 µM αS	<0.0001	****	t=13.28, df=121.3
**tau_{PRR}**	**P value**	**P value significance**	**t, df**
+ 50 µM αS	<0.0001	****	t=11.64, df=132.1
+ 100 µM αS	<0.0001	****	t=11.81, df=107.2
+ 150 µM αS (top population)	<0.0001	****	t=27.34, df=60.13
+ 150 µM αS (bottom population)	<0.0001	****	t=10.14, df=38.20
+ 150 µM $αS_{1-100}$	0.3998	ns	t=0.2545, df=119.4
**tau_{PRR}**	**P value**	**P value significance**	**t, df**
+ 50 µM αS	<0.0001	****	t=5.498, df=126.6
+ 50 µM $αS_{pS129}$	0.0003	***	t=3.564, df=117.9
+ 100 µM αS	<0.0001	****	t=9.228, df=144.6
+ 100 µM $αS_{pS129}$	<0.0001	****	t=5.890, df=127.6
+ 150 µM αS	<0.0001	****	t=9.189, df=138.6
+ 150 µM $αS_{pS129}$	<0.0001	****	t=5.595, df=158.2
**eGFP**	**P value**	**P value significance**	**t, df**
+ 150 µM αS	<0.0001	****	t=4.976, df=147.5

S2 Table. Results of applying one-tailed t-tests. Results of one-tailed t-tests (GraphPad Prism) for tau binding to monomer αS, ns = not significant, t = t value and df degrees of freedom with a Geisser-Greenhouse correction.
