# Supplementary material for: Multivalency drives interactions of alpha-synuclein fibrils with tau
Source: PLoS One. 2024 Sep 10;19(9):e0309416. doi: 10.1371/journal.pone.0309416 (PMC11386428; doi:10.1371/journal.pone.0309416)
Supplement: S3 Table — Classification of τD2app for tau binding to αS seeds as described in the main manuscript. Type I: τD2< 1.6*τD1; Type II: τD2>10 ms; Type III: 1.6* τD1<τD2<10 ms. (PDF) [file pone.0309416.s013.pdf]

Tau construct (+ αS seed)	Type I (%)	Type II (%)	Type III (%)
**tau$_{1N4R}$**	-	-	-
20 nM αS	47	12	41
60 nM αS	21	5	74
100 nM αS	15	23	62
20 nM αS$_{1-100}$	60	17	23
60 nM αS$_{1-100}$	69	13	18
100 nM αS$_{1-100}$	40	13	47
**tau$_{4R}$**	-	-	-
20 nM αS	39	12	49
60 nM αS	6	3	41
100 nM αS	23	11	66
20 nM αS$_{1-100}$	62	12	26
60 nM αS$_{1-100}$	36	4	60
100 nM αS$_{1-100}$	39	12	49
**tau$_{PRR}$**	-	-	-
20 nM αS	41	16	43
60 nM αS	0	1	99
100 nM αS	0	3	97
20 nM αS$_{1-100}$	46	12	42
60 nM αS$_{1-100}$	36	10	54
100 nM αS$_{1-100}$	19	21	60
**tau$_{PRR}$**	-	-	-
60 nM αS (5 mM NaCl)	0	1	99
60 nM αS (50 mM NaCl)	2	5	93
60 nM αS (500 mM NaCl)	45	9	46
**eGFP**	-	-	-
60 nM αS	9	26	65

S3 Table. Classification of seed diffusion times. Classification of τ_{D2app} for tau binding to αS seeds as described in the main manuscript. Type I: τ_{D2}< 1.6*τ_{D1}; Type II: τ_{D2}>10 ms; Type III: 1.6* τ_{D1}<τ_{D2}<10 ms.
