# Supplementary material for: Multivalency drives interactions of alpha-synuclein fibrils with tau
Source: PLoS One. 2024 Sep 10;19(9):e0309416. doi: 10.1371/journal.pone.0309416 (PMC11386428; doi:10.1371/journal.pone.0309416)
Supplement: S4 Table — Quantification of binding of tau1N4R, tau4R and tauPRR with increasing concentrations of αS monomer. Mean τD, norm and SD calculated for a minimum of three measurements for each concentration αS. % diff is calculated as [τD(+αS)-τD(-αS)]/τD(-αS). (PDF) [file pone.0309416.s014.pdf]

construct	$\tau_{D, norm}$	% diff
tau_{1N4R}	1.00±0.042	-
+ 150 μM αS	1.07±0.054	7
tau_{4R}	1.00±0.043	-
+ 50 μM αS	1.02±0.047	2
+ 100 μM αS	1.05±0.043	5
+ 150 μM αS	1.10±0.050	10
tau_{PRR}	1.00±0.040	-
+ 50 μM αS	1.06±0.038	6
+ 100 μM αS	1.08±0.050	8
+ 150 μM αS(top population)<br>+ 150 μM αS(bottom population)	1.27±0.067<br>1.07±0.030	27<br>7
+ 150 μM αS_{1-100}	1.00±0.043	0

S4 Table. Quantification of binding at 150 μM αS monomer. Quantification of binding of tau_{1N4R}, tau_{4R} and tau_{PRR} with increasing concentrations of αS monomer. Mean $\tau_{D, norm}$ and SD calculated for a minimum of three measurements for each concentration αS. % diff is calculated as $[\tau_D(+\alpha S)-\tau_D(-\alpha S)]/\tau_D(-\alpha S)$.
