# Supplementary material for: Multivalency drives interactions of alpha-synuclein fibrils with tau
Source: PLoS One. 2024 Sep 10;19(9):e0309416. doi: 10.1371/journal.pone.0309416 (PMC11386428; doi:10.1371/journal.pone.0309416)
Supplement: S5 Table — Summary median type III τD2app, fB (with associated quartiles, 25% & 75%), and Q values for eGFP and for tauPRR in low salt (5 mM NaCl), standard salt (50 mM NaCl), and high salt (500 mM NaCl) conditions. Minimum of three replicates for each. (PDF) [file pone.0309416.s015.pdf]

construct	τ_{D2app}	f_B	Q
eGFP	-	-	-
+ 60 nM αS	3.7 (1.8, 5.4)	0.022 (0.015, 0.044)	1.0
tau_{PRR}	-	-	-
+ 60 nM αS (5 mM NaCl)	2.2 (1.6, 3.6)	0.50 (0.42, 0.62)	3.6
+ 60 nM αS (50 mM NaCl)	3.0 (2.4, 4.5)	0.22 (0.17, 0.32)	1.0
+ 60 nM αS (500 mM NaCl)	2.0 (1.3, 4.4)	0.031 (0.011, 0.053)	1.0

S5 Table. τ_{D2app}, Q and f_B for GFP and PRR salt concentrations. Summary median type III τ_{D2app}, f_B, along with associated quartiles (25, 75%), and Q values for eGFP and for tau_{PRR} in low salt (5 mM NaCl), standard salt (50 mM NaCl), and high salt (500 mM NaCl) conditions. Minimum of three replicates for each.
