# Supplementary material for: Multivalency drives interactions of alpha-synuclein fibrils with tau
Source: PLoS One. 2024 Sep 10;19(9):e0309416. doi: 10.1371/journal.pone.0309416 (PMC11386428; doi:10.1371/journal.pone.0309416)
Supplement: S6 Table — Classification of τD2app for tau1N4R binding to αS in solutions with high concentrations of tau1N4R (i.e. comparable to those used for tau1N4R aggregation). Type I: τD2< 1.6*τD1; Type II: τD2>10 ms; Type III: 1.6* τD1<τD2<10 ms. (PDF) [file pone.0309416.s016.pdf]

Tau construct	Type I (%)	Type II (%)	Type III (%)
**tau_{1N4R}**	-	-	-
1 μM αS seed, 4.98 μM tau	46	21	33
1 μM $αS_{1\text{-}100}$ seed, 4.98 μM tau	69	12	19

S6 Table. Classification of diffusion times with seeded αS/tau aggregation FCS conditions.

Classification of τ_{D2app} for tau_{1N4R} binding to αS in solutions with high concentrations of tau_{1N4R} (i.e. comparable to those used for tau_{1N4R} aggregation). Type I: $\tau_{D2} < 1.6 * \tau_{D1}$; Type II: $\tau_{D2} > 10$ ms; Type III: $1.6 * \tau_{D1} < \tau_{D2} < 10$ ms.
