# Supplementary material for: Multivalency drives interactions of alpha-synuclein fibrils with tau
Source: PLoS One. 2024 Sep 10;19(9):e0309416. doi: 10.1371/journal.pone.0309416 (PMC11386428; doi:10.1371/journal.pone.0309416)
Supplement: S1 Raw image — (PDF) [file pone.0309416.s017.pdf]

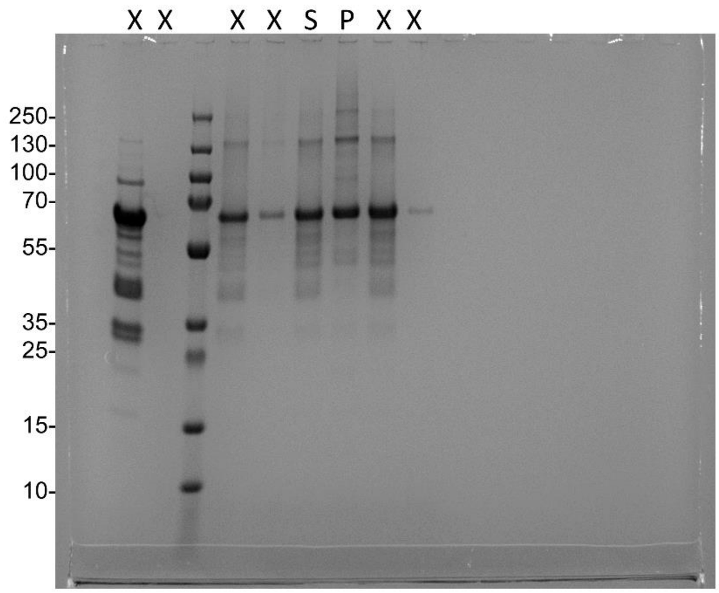

- Original uncropped SDS-PAGE gels of Figure 4c for **tau**$_{\mathbf{1N4R}}$ samples post-aggregation with S indicating the supernatant and P the pellet after centrifugation
- The indicated S and P lanes are for the 1st **tau**$_{\mathbf{1N4R}}$ panel in Figure 4c
- The amount for each sample was using the relative amount of pellet vs. the total amount of pellet and supernatant (ImageJ) for Figure 4d
- SDS-PAGE gels were imaged on a G:BOX mini gel imager

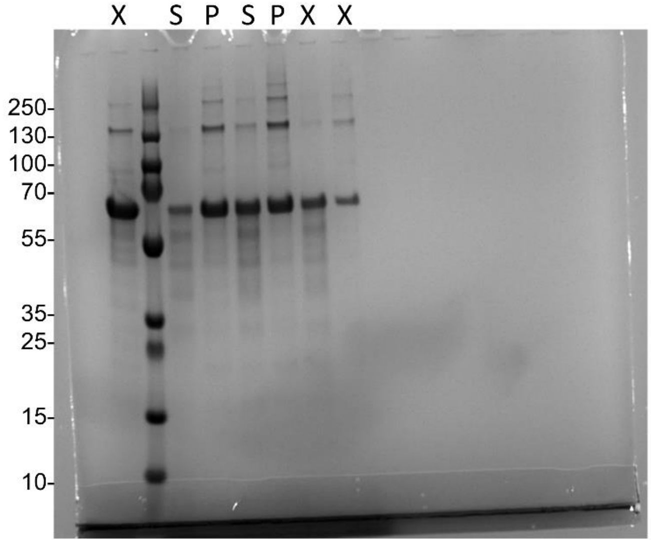

- Original uncropped SDS-PAGE gels of Figure 4 for **tau**$_{\mathbf{1N4R}}$ samples post-aggregation with S indicating the supernatant and P the pellet after centrifugation
- The indicated S and P lanes are for the 2nd and 3rd **tau**$_{\mathbf{1N4R}}$ panels in Figure 4c
- The amount for each sample was using the relative amount of pellet vs. the total amount of pellet and supernatant (ImageJ) for Figure 4d
- SDS-PAGE gels were imaged on a G:BOX mini gel imager

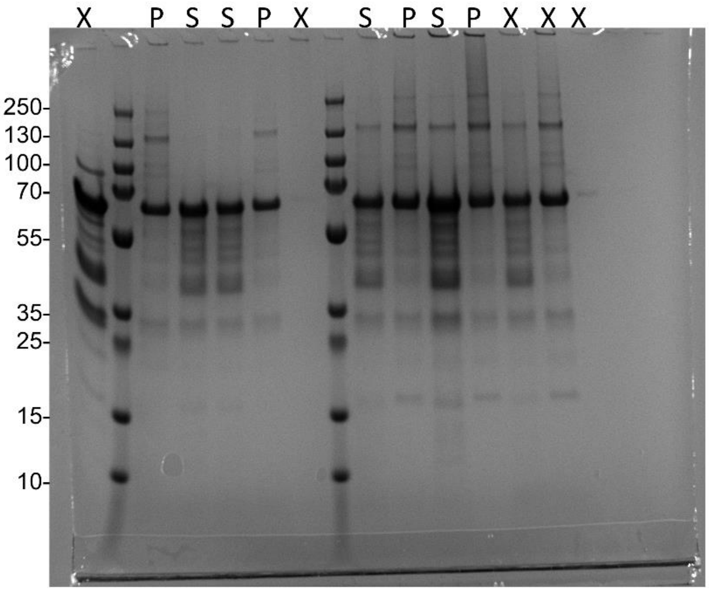

- Original uncropped SDS-PAGE gels of Figure 4 for **tau$_{1N4R}$ + αS seeds** and **tau$_{1N4R}$ + αS$_{1\text{-}100}$ seeds** samples post-aggregation with S indicating the supernatant and P the pellet after centrifugation
- The first two S P sets from left to right is the **tau$_{1N4R}$ + αS$_{1\text{-}100}$ seeds** 1st and 2nd panels (P comes first due to mistake while loading) for Figure 4c
- The next two sets after the ladder in the middle is the **tau$_{1N4R}$ + αS seeds** 1st and 2nd panels for Figure 4c
- The amount for each sample was using the relative amount of pellet vs. the total amount of pellet and supernatant (ImageJ) for Figure 4d
- SDS-PAGE gels were imaged on a G:BOX mini gel imager

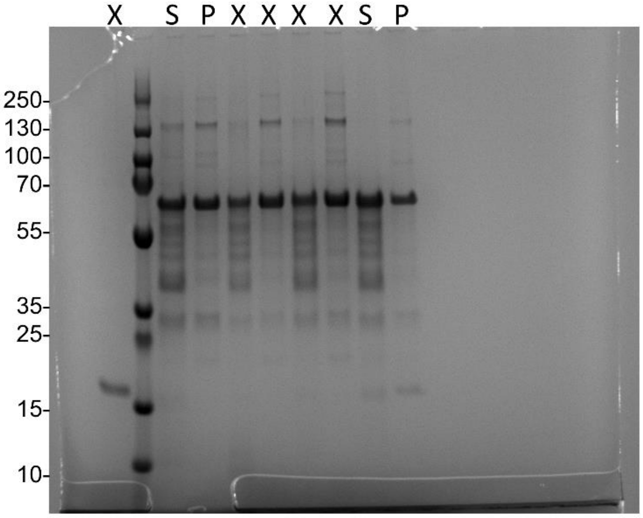

- Original uncropped SDS-PAGE gels of Figure 4 for **tau$_{1N4R}$ + αS seeds** and **tau$_{1N4R}$ + αS$_{1\text{-}100}$ seeds** samples post-aggregation with S indicating the supernatant and P the pellet after centrifugation
- The first S P set from left to right is the **tau$_{1N4R}$ + αS$_{1\text{-}100}$ seeds** 3rd panel, the right-most S P set is **tau$_{1N4R}$ + αS seeds** 3rd panel for Figure 4c
- The amount for each sample was using the relative amount of pellet vs. the total amount of pellet and supernatant (ImageJ) for Figure 4d
- SDS-PAGE gels were imaged on a G:BOX mini gel imager

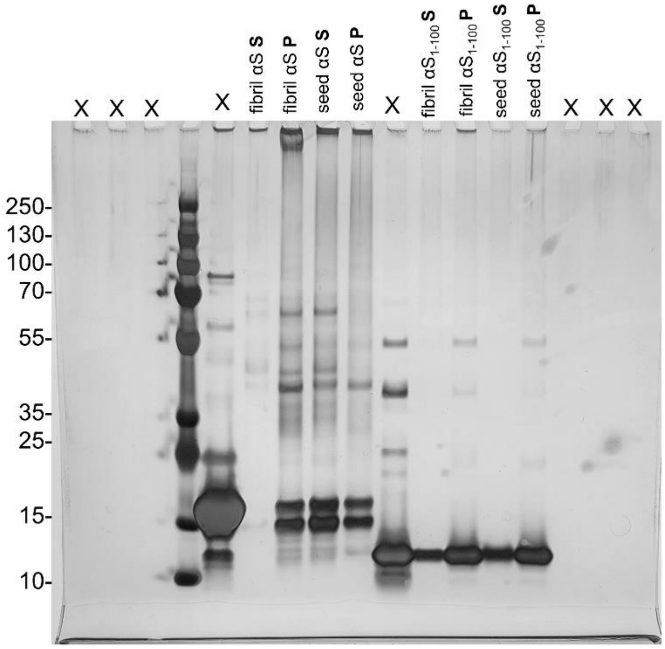

- Original uncropped SDS-PAGE gels of S2 Fig c for **αS fibrils and αS_{1-100} fibrils versus αS seeds and αS_{1-100} seeds** with S indicating the supernatant and P the pellet after centrifugation
- The amount for each sample was using the relative amount of pellet vs. the total amount of pellet and supernatant (ImageJ) for S2 Fig d
- SDS-PAGE gels were imaged on a G:BOX mini gel imager

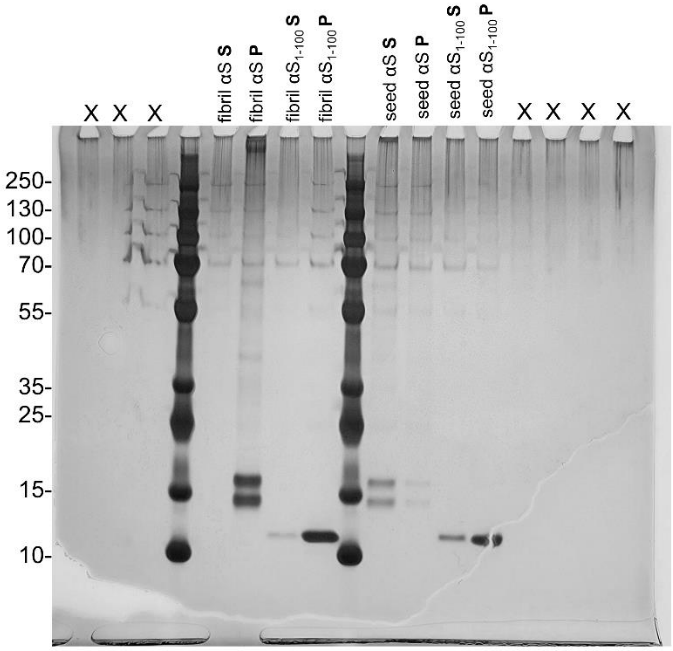

- Original uncropped SDS-PAGE gels of S2 Fig c for **αS fibrils and αS_{1-100} fibrils versus αS seeds and αS_{1-100} seeds** with S indicating the supernatant and P the pellet after centrifugation
- The amount for each sample was using the relative amount of pellet vs. the total amount of pellet and supernatant (ImageJ) for S2 Fig d
- SDS-PAGE gels were imaged on a G:BOX mini gel imager
