# Supplementary material for: Multivalency drives interactions of alpha-synuclein fibrils with tau
Source: PLoS One. 2024 Sep 10;19(9):e0309416. doi: 10.1371/journal.pone.0309416 (PMC11386428; doi:10.1371/journal.pone.0309416)
Supplement: Attachment — Submitted filename: Response to Reviewers.docx [file pone.0309416.s018.docx]

Addressing Reviewers’ and Editor’s comments

We extend our sincere gratitude to the reviewers and the Editor for dedicating their time and effort to reviewing our manuscript and providing us with valuable constructive feedback. We have thoroughly addressed all the comments from the reviewers and are confident that our response has resulted in substantial improvements to our manuscript.

Editor Comments:

Journal Requirements:

When submitting your revision, we need you to address these additional requirements.

1. Please ensure that your manuscript meets PLOS ONE's style requirements, including those for file naming. The PLOS ONE style templates can be found at

[https://journals.plos.org/plosone/s/file?id=wjVg/PLOSOne_formatting_sample_main_body.pdf](https://urldefense.com/v3/__https:/journals.plos.org/plosone/s/file?id=wjVg*PLOSOne_formatting_sample_main_body.pdf__;Lw!!IBzWLUs!XNnfrbClmu91pQrTGoacU1BxaO4rsIpMtbGVLUGJH3rGnzSP4B_VQpQ1FDFy9spBIDjD2SQ6Y-f2dzNejJz-1Yy3SKS8UaGcFiGvwfF_$) and 
[https://journals.plos.org/plosone/s/file?id=ba62/PLOSOne_formatting_sample_title_authors_affiliations.pdf](https://urldefense.com/v3/__https:/journals.plos.org/plosone/s/file?id=ba62*PLOSOne_formatting_sample_title_authors_affiliations.pdf__;Lw!!IBzWLUs!XNnfrbClmu91pQrTGoacU1BxaO4rsIpMtbGVLUGJH3rGnzSP4B_VQpQ1FDFy9spBIDjD2SQ6Y-f2dzNejJz-1Yy3SKS8UaGcFk22qJpP$)

**We have reviewed the PLOS ONE style requirements and have updated our manuscript and file naming to reflect this.**

2. We note that the grant information you provided in the ‘Funding Information’ and ‘Financial Disclosure’ sections do not match.

When you resubmit, please ensure that you provide the correct grant numbers for the awards you received for your study in the ‘Funding Information’ section.

**We have ensured the correct grant numbers are now provided.**

Additional Editor Comments:

The manuscript in its current form fails to comply with multiple of the PLOS ONE publication criteria ([https://journals.plos.org/plosone/s/criteria-for-publication](https://urldefense.com/v3/__https:/journals.plos.org/plosone/s/criteria-for-publication__;!!IBzWLUs!XZqVYCQN5ytpiEHEi88EOTNVVTYsUOFYRAxB4vigWjVeS8aoRQRQiUmBcNpFeHbEwIWpmmHRm31-dfFQPsx7jwXrXGM$)). In particular, it fails to meet criterion 3, based on incomplete information on the experimental conditions, a lack of sufficient experimental conditions (and replicates) and concerns about the experimental design more generally. These concerns affect multiple parts of the experiments in the manuscript (see reviewer comments). In addition, there are concerns that the provided data do not fully support the conclusions (and title) of the manuscript (i.e. criterion 4). Although PLOS ONE does not require novelty, it is important that the manuscript properly addresses and discusses previous related publications (criterion 2). You can find a further delineation of concerns, and a request for additional data to be provided, in the reviewer comments. I generally agree with the concerns raised by the reviewers, and we will require a thorough revision of the manuscript (and the data contained therein) and a detailed response to each point raised by the reviewers.

**We have updated our methods section to include the number of replicates for each experiment. We have also deposited all our raw FCS curves and aggregation data on figshare https://doi.org/10.6084/m9.figshare.c.7228426.v1. We also included references and discussions pertaining to relevant previous publications in our revised discussion.**

Aside from the comments from the reviewers, please also address the following concerns I have:

- Figure 4: I assume different lanes in the SDS PAGE gel are meant to be replicates but they seem to differ a lot. This should be explained/discussed. I would actually advise that this should be repeated, unless I am misunderstanding the nature of the lanes in this gel. (in which case this should be better explained in the caption)

**Yes, this is correct that these were meant to be replicates, but that we had issues with consistency of the samples in the previous version of the manuscript, as seen in this figure. We have since revised our aggregation protocol as described in the Materials &Methods, resulting in both reproducible aggregation kinetics, as well as in reproducibility in our SDS-PAGE analysis of the proteins in the supernatant and pellet following completion of the kinetic measurements. The original Figure 4 has been replaced with entirely new data in the current version of Figure 4.**

- Figure 5: currently the manuscript just notes that 'fibrillar aggregates' are still formed, but does not discuss morphological changes that are apparent in the EM. This needs to be rectified, as morphological changes are indicative of changes in the aggregation mechanism induced by aSYN seeds.

**We agree that there are differences in the morphology of the fibrillar aggregates but believe any in depth interpretation or discussion of this is beyond the scope of this manuscript. The purpose of this figure is simply to confirm that we are forming fibrillar aggregates under all conditions and not amorphous assemblies. In response to Reviewer 2, we have moved the figure with the TEM images to the Supplement.**

- Figure 7 seems to show (unexpected?) inhibitory effects of the aSYN aggregates at the highest concentration. This needs to be discussed in the manuscript.

**We now include kinetic traces in the revised Figure 4 which confirm that both full-length and αS_1-100_ seeds inhibit tau_1N4R_. These traces and their analysis are now included in Figure 4. We have updated our discussion of the inhibitory effect of the αS aggregates, including a citation of prior reports of this phenomenon.**

As noted by the reviewers, the current data do not fully support the conclusions, due to limitations in the data and results. Unless these concerns are fully and carefully addressed, I am afraid that the manuscript would not be suitable for publication in PLOS ONE. I therefore stress the importance of a thorough and major revision. Particular attention needs to be paid to the characterization of the seeds, a more detailed analysis of time-dependent aggregation kinetics, a discussion of (and perhaps increased use of) replicates in the various experiments, statistical analysis, FCS analysis, and better explanation of the 'multivalency' evidence from the current study.

**We thank the editor for this summary and have addressed each of these in our response to the reviewers below.**

Comments Reviewer 1:

The paper studies the interactions between full-length tau and individual tau domains with monomeric and aggregated α-synuclein (αS) using FCS, ThioflavinT, TEM, and charge analysis. The findings suggest a nuanced interplay requiring multivalency, challenging the simplistic notion of direct interaction between tau and alpha-synuclein. The paper addresses a topic of high importance for understanding the origins of mixed pathologies in neurodegenerative disorders. The paper contributes valuable insights into tau-αS interactions, but the authors should address the concerns below to make it suitable for publication.

The conclusion that αS does not affect tau aggregation is questionable due to the relatively coarse time points of ThT fluorescence measurements (every 8-10 hours), potentially missing crucial aggregation windows. The authors should repeat data collection with finer time intervals (10-15 mins) to strengthen the conclusions.
**We thank the reviewer for this suggestion. We completely revised our aggregation protocol to allow for real-time monitoring of the aggregation kinetics on a Tecan Spark plate reader which are now shown in Figure 4. While our prior analysis focused only on the amount of tau converted to aggregate at the final time-point in our assay, our new protocol allows us to compare the kinetics of aggregation as well. Interestingly, this leads us to observe that the presence of αS aggregates delays aggregation of tau and confirms our prior finding that the total tau converted to aggregates is not impacted by the αS aggregates. Figure 4 consists of completely new data and we believe allows us a more nuanced discussion of the impact of αS aggregates on tau aggregation.**

The authors should include paragraph(s) in FCS and ThT section of the Methods detailing the number of technical and biological replicates used for their studies.

**The FCS measurements include at minimum three technical replicates per construct at its respective concentration. The aggregations include three technical replicates per aggregation condition and the aggregations overall themselves are three technical replicates. We have included this information in the Methods section as recommended.**

The FCS findings are likely dampened due to the heterogeneity of the solutions authors used as seeds. However, generating heterogeneous samples containing a discrete size of oligomers is quite challenging. The authors need to include a paragraph in the Discussion addressing the effect sample heterogeneity on the interpretation of FCS data.
**We agree with the reviewer that the seeds used are a heterogenous mixture of monomer, oligomer and fibrillar αS and this is now described explicitly in the text. While our discussion of the FCS measurements the presence of the seeds already addressed the heterogenous nature of the interaction, we have added further explanation in both the Materials and Methods and the Discussion to try to clarify any remaining points of confusion.**

Minor suggestions
1) Add a paragraph in the introduction discussing current in vivo models of synuclein and tau

**We have added a few sentences in the introduction discussing *in vivo* models.**

2) In Fig. 1, provide diagrams of all constructs (tauPRR, tau4R, and tau1N4R) used in the study along with the location of fluorescent tag (tau)

**We have updated Figure 1 to reflect this suggestion.**

3) In Fig. 1 for synuclein, display the different constructs and label the acetylation and phosphorylation sites.

**To again address comments 2 and 3, in our revised manuscript, Figure 1 now contains a schematic of construct. Locations of the N-terminal acetylation, phosphorylation and fluorescent tags are indicated on the relevant schematics.**

3) Fig 2, 3 – in the figure captions, add the concentrations of tau constructs and the molar ratios of tau: syn

**Both Figure 2 and 3 captions were updated to include this information.**

4) Similar to synuclein, PTMs are implicated in tau aggregation. Add a paragraph explaining the rationale for not using tau with PTM in the results.

**Researching tau post-translational modifications (PTMs) has posed significant challenges due to the complexity of generating tau with authentic PTMs. Our study primarily aimed to investigate the domains of tau responsible for its interaction with αS, rather than dissection of the roles of PTMs in this interaction. We believe that a paragraph addressing tau PTMs would distract from the focus of the manuscript.**

5) The quality of TEM micrographs should be revisited – it was difficult to assess whether the low quality is due to the file conversion or the actual data

**We have included new TEM micrographs and have moved this figure to the Supplement.**

6) The findings of Figs S3 are important and should be moved to the main Fig 2

**The binding exhibited a weak concentration-dependent pattern for tau_4R_ vs tau_PRR_, so we do not consider it to contribute significantly to the main message conveyed in Figure 2.**

7) Figure S7 should be added as a panel in Fig. 5.

**We moved our updated aggregation plots to Figure 4.**

Comments Reviewer 2:

The manuscript entitled „Multivalency drives interactions of alpha-synuclein fibrils with tau” presents an interesting set of experimental results that could contribute to better understanding of development, prevention and future therapies of neurodegenerative diseases, such as Alzheimer’ disease and Parkinson’s disease. The text is well and clearly written. The conclusions of the work are based mainly on the fluorescence correlation spectroscopy (FCS). The method is related to the hydrodynamic properties of fluorescently labeled species that diffuse freely through a small focal volume. The methodological approaches are described in much detail. The laboratory work is well done, with almost all necessary strict controls (some details are discussed below). Having said that, however, I would have some serious concerns that would need to be clarified in a revised version of the manuscript.

Major concerns:

1. The motivation to undertake studies of the N-terminal alpha-S 100 residues is unclear. The Authors inform us in the Introduction section that the C-terminal part is important for tau binding, while the N-terminal residues are involved in mediation of binding to lipid bilayers. A scientific question needs to be clearly addressed regarding the putative interaction of the N-terminal fragment of alpha-S with tau in the context of membrane binding

**We are somewhat confused by this comment.** **We note that we do not address αS membrane binding in this manuscript and do not believe we need to explicitly address simultaneous binding of αS with membrane and tau. αS binds dynamically and reversibly to membranes. Even in cells, a significant fraction of αS is cytoplasmic. Thus, αS may interact with binding partners in the cytoplasm independent of its interaction with membranes. In the Introduction to the manuscript, we highlight prior studies which have demonstrated the significance of the αS C-terminus in binding to tau through dot blot assays and spin-down assays (reference 20). Therefore, our work had the goal of further examining the role of αS’s C-terminus in interactions with tau, while simultaneously investigating which domains of tau mediate this interaction using biophysical techniques. Investigating the interaction of the N-terminus of αS with tau in the context of membrane binding was not the focus of our study.**

2. The final conclusions seem to confirm the title "multivalence" only indirectly. In my opinion, the title seems to be a bit too far-reaching compared to the results described in this paper. The results and conclusions should address the multivalency more unambiguously.

**We respectfully disagree with the reviewer’s comment. αS monomer has only weak interactions with tau, but αS fibrils have interactions detectable in the nM regime. Experiments with truncated seeds indicate that the C-terminus plays a role, particularly with full-length tau, but that other αS regions also play a role. These interactions appear to be primarily electrostatic in nature. Therefore, the interactions of tau with full-length fibril seeds are presumably not the result of a well-folded domain in the fibrils, but rather a result of the multi-valent display of charged functional groups in αS fibrils/seeds. Additionally, from the tau perspective, our FCS results illustrate that both the MTBR and the PRR serve as bindings sites for tau to αS monomer. The diffusion time distribution of the PRR in the presence of monomer αS shows two peaks, illustrating that there are at least two binding sites for αS in the PRR alone. Lastly, analysis of the brightness parameter, Q, in the binding of tau to αS seeds demonstrates multiple tau associated with the seeds. All of these underscore the importance of multivalency in the interactions between tau and αS monomer and seeds. As suggested by the reviewer, we have edited the text of the manuscript to address multivalency more unambiguously.**

3. The FCS approach links the hydrodynamics with spectroscopy, and hence to analyze the hydrodynamic properties in the exact, quantitative manner, it is necessary to take into account the photophysical properties of the fluorescent probe (Alexa Fluor triplet state in this case) together with the diffusive properties of the studied species. Alternatively, the Authors should prove directly that neglecting of the Alexa Fluor triplet state (with the characteristic life-time in the range of microseconds) does not influence the diffusion time determination. Moreover, it is recommended to fit a two-component equation to FCS autocorrelation curves even for a single protein species to check for the purification level from the residual dye present in the solution after labeling, that could significantly change the apparent results.

**We agree with the reviewer’s comments and note that in the almost two decades that we have been using FCS to quantify binding interactions between fluorescently labeled proteins and their partners, we are keenly aware that both free dye and high laser powers for excitation resulting in triplet state kinetics of the fluorophore can complicate analysis. As we have previously published (*J. Biol. Chem.* (2019):294(50) 19381–19394; *J. Am. Chem. Soc.* (2012), 134, 40, 16607–16613;** ***Biochemistry* (2009), 48, 11, 2304–2306; the laser powers we use for excitation (5 uW prior to entry into the microscope) does not cause any triplet state in Alexa 488 fluorophore. Furthermore, we have rigorous protocols for removal of free dye and evidence of our competence in this matter can be seen by the fact that the diffusion times measured for proteins show batch to batch consistency; if trace amounts of free dye were present, we would expect variability in the measured diffusion time. We now include text in the Materials and Methods to further explain our protocols relating to this point and a figure comparing the following fits: single diffusing component, single diffusing with triplet state component, two diffusing components. An F-test demonstrates that the additional free parameters in the latter two fits are not justified. This is included as S3 Fig.**

4. How did the Authors determine, control or check the dimensions or the monomeric/oligomeric/fibril composition of the heterogeneous seed solutions after sonication and the repeatability of the process?

**In the original submission, TEM imaging after aggregation showed the fibril structure of material used as seeds. We have now analyzed both our full-length and truncated seeds by SDS-PAGE and included this as a supplemental figure (S2 Fig). This demonstrates that our ‘seeds’ contain mixtures of fibrillar, oligomer and monomer αS. We have also included a post-sonication image of our full-length ‘seeds’ by TEM (S2 Fig).**

5. The TEM images are below publication quality and should be moved to the Supplement, in particular since they do not contribute significantly to the conclusions.

**We have included new TEM micrographs and per the reviewer’s suggestion they have been moved to the supplement (S8 Fig). In our experience, obtaining high quality TEM images of tau aggregates is challenging (for example, as compared to αS, with which we also have experience). However, as the major role of these images is to provide support for the formational of fibrillar aggregates rather than amorphous assemblies, and not for detailed analysis of the structures of the fibers formed, we believe our new images clearly demonstrate fibrillar aggregate formation.**

6. My main concern is, however, related to the statistical data analysis and their interpretation. While the use of the relative diffusion time values derived from FCS is a very good idea, this should be performed with care. First of all, this is only a semi-quantitative “fingerprint” of the aggregation process. The autocorrelation FCS curve, as defined in this work, is related to such a number of diffusing species at equilibrium as defined by the n-component equation. These conditions are not met in the case of aggregation, since (1) the resulting aggregates are heterogeneous (of many different possible types) and (2) they appear during the diffusion time through the focal volume (non-equilibrium state). In particular, the oscillating “serrated pattern” at the longer lag times in S2 Fig. shows this clearly. In consequence, the relative diffusion time values (and the remaining fitting parameters) should be denoted to as “apparent”, since they cannot be ascribed to any given well defined protein complex or state. Moreover, the next question is whether the quantitative statistical parameters such as: median, mean and standard deviation are even applicable to these data, since each data point comes from its own autocorrelation curve with its own uncertainty, which is sometimes tiny, and sometimes huge. Thus, such points cannot be treated as equivalent input for averaging. In my opinion, such a seemingly strict, quantitative statistical analysis is illegitimate and FCS data should be treated only semi-quantitatively as an illustration of the analyzed process.

**We agree with the reviewer’s perspective that the FCS analysis is semi-quantitative and have taken care not to overinterpret the results based on our analysis. We do not use the diffusion times to draw any conclusions about the sizes of the diffusion species and have revised the text and legends to use an apparent diffusion time in response to this reviewer comments. Additionally, some of the reviewer’s concern appears to be misunderstanding. The curves showing the oscillating patterns in S4 Figure are examples of curves that were discarded from our analysis because of our inability to analyze them meaningfully – i.e. the curves that the reviewer points out as problematic were never included in any of the analysis. This is a relatively small fraction of curves that are discarded, as reported in Table S1. We have edited our description of how curves are discarded to make this more clear. There also appears to be some misunderstanding regarding the degree of uncertainty in the diffusion times measured: because we use goodness of fit as a criteria for selecting curves to be discarded, while there is certainly some distribution in the uncertainty in the fit parameter values, it is not ‘sometimes tiny, and sometimes huge’. For the diffusion times with Gaussian distributions (illustrated in S7 Fig), it is certainly appropriate to use the mean value as a point of comparison. For non-symmetric distributions, which have long tails at higher diffusion times (illustrated in S7 Fig), the mean is not appropriate. The median is the preferred measure of the central tendency of asymmetric distributions and is thus the most appropriate value for comparative purposes of these distributions. To address this, we have revised the legends to designate the diffusion times as “apparent,” thereby clarifying their interpretative nature.**

Minor concerns:

1. Some fragments of the Methods section are repeated, in particular most of the protein expression and purification protocols are common for all constructs. The Authors may consider shortening of the text. Moreover, some buffer solutions of the same composition are denoted to as different names, eg. TBB and ASB3. The nomenclature should be made consistent throughout the text.

**The purification methods for full-length alpha-synuclein and the C-terminally truncated construct are distinct due to their derivation from different vectors. For ASB3, the correct buffer composition is 50 mM HEPES, 300 mM imidazole, pH 7.5. We appreciate the reviewer’s observation in identifying this typographical error, which has since been rectified.**

2. Was 10% glycerol used for freezing the protein samples?

**No**

3. In p. 7, top, the extinction coefficients should be written in a more eligible way: Eps 280 nm = 7450 and 1490 M-1cm-1 for tau 1N4R and tau4R/PRR, respectively.

**This has been fixed in the manuscript.**

4. There are some typos, such as “alhough” in p. 5, and repeats “in our”, “set of curves”. The text should be polished.

**The indicated typographical errors have been fixed and the entire manuscript has been proof-read.**

5. The MALDI mass spectrum is unreadable. The figure should display clearly the deconvoluted spectrum, with the readable axes legends and numbering.

**The mass spectrum was re-plotted to make it more legible.**

6. The UV spectrum should be equipped with its own legend. This is not a part of the MALDI spectrum. The elevated values of absorbance in the range above 310 nm suggest the presence of the protein aggregates. This should be explained.

**The UV spectrum is accompanied by its own legend within the figure. We have revised the associated figure caption to enhance clarity and distinguish between the UV spectrum and the MALDI spectrum. The very low extinction coefficients of these proteins means that relatively high concentrations are needed in order to reliably determine the concentration of the protein by absorbance at 280 nm. The very slight increase in the baseline leading up to the aromatic absorbance appears to be exaggerated because of this and is consistent with what is observed for globular proteins with higher extinction coefficients – it just appears as a larger contribution due the low contribution at 280 nm. We do not see any evidence for any significant contribution from aggregates in the monomer proteins samples by FCS.**

Comments Reviewer 3:

The authors investigate interaction between tau and alpha-synuclein (αSyn) proteins associated with neurodegenerative diseases. Using fluorescence correlation spectroscopy, the authors found that tau interacts weakly with monomeric αSyn, but that this interaction is much more pronounced for αSyn aggregates. They also found that the interaction between tau and αSyn does not significantly impact tau aggregation or fibril formation. The authors suggest that the weak and transient interactions between tau monomers and αSyn seeds may act to buffer the monomer tau concentration rather than provide a surface to template tau aggregation.
Although the use of fluorescence correlation spectroscopy techniques to study tau and αSyn interactions is interesting, the findings provide little new information: it is known that both tau and αSyn independently and together form condensates that transition into aggregates.
Major concerns are:
The Materials and Method section on page 9 describes seeds as “seeds’ are expected to be a mix of monomer, oligomer and fiber”, but no characterization has been performed to confirm this assumption.

**From our response to Reviewer 2: We have now analyzed both our full-length and truncated seeds by SDS-PAGE and included this as a supplemental figure. This demonstrates that our ‘seeds’ contain a mixture of fibrillar, oligomer and monomer αS.**

There are numerous evidences supporting the concept that αSyn aggregates or seeds can promote tau aggregation in vitro, cell culture studies and in vivo (J. Biol. Chem. (2020) 295(21) 7470 –7480; Biological Psychiatry 2018, 84, 499-508; Cell. 2013,154(1):103-17. Sci Rep 12, 2987 (2022); J Exp Med (2021) 218 (1): e20192193.). However, this study did not see acceleration of tau aggregation in the presence of αS seeds. This study relies solely on ThT assay for the aggregation experiments. The SDS-PAGE assay was performed using a single set of conditions in which 1 μM of αSyn seed was mixed with tau1N4R at a 1:5 molar ratio of αSyn to tau1N4R to study the aggregation assay. However, it is essential to conduct further studies on the aggregation using different sets of conditions. In addition to ThT and SDS-PAGE, other assays such as native gel, etc., need to be included to analyze aggregation studies.
In summary, the data seem incomplete, provide not enough novelty, and do not support the conclusion.

**We include in Figure 4 an entirely new set of data which includes kinetic traces of tau aggregation, along with gel analysis of the amount of aggregated material. While our prior analysis focused primarily on the amount of tau converted to fibrillar material at the completion of aggregation, our new experiments allow us to compare both this parameter as well as aggregation kinetics. We note that we find, somewhat unexpectedly, that under the conditions of our measurements, the presence of αS seeds actually inhibits aggregation of tau.** **This is consistent with other published results and our edited discussion includes both reference to these publications** (**J Biol Chem. 2020;295(21):7470-80.) and (J Biol Chem. 2016;291(29):15046-56.) as well as the ones** **which show enhancement of tau aggregation** **listed above by the reviewer. We note that the use of ThT and SDS-PAGE analysis is commonly used to characterize aggregation of amyloid proteins; native gel may potentially tell us more about the distribution of the aggregate sizes, but given that structural characterization of the aggregates is not of relevance to our current work, we consider it beyond the scope. We also want to emphasize that aggregation can be highly variable, depending upon salt, buffer, speed of mixing/shaking, concentration of protein, the presence of molecular aggregation inducers (such as heparin) etc. In our case, it was most important to be able to characterize the impact of αS seeds on tau aggregation complementary to that used for our FCS studies.**
