# Supplementary material for: Multivalency drives interactions of alpha-synuclein fibrils with tau
Source: PLoS One. 2024 Sep 10;19(9):e0309416. doi: 10.1371/journal.pone.0309416 (PMC11386428; doi:10.1371/journal.pone.0309416)
Supplement: Attachment — Submitted filename: Response to Reviewers.docx [file pone.0309416.s019.docx]

Addressing Reviewers’ and Editor’s comments

We extend our sincere gratitude to the reviewers and the Editor for dedicating their time and effort to reviewing our manuscript and providing us with valuable constructive feedback. We have thoroughly addressed all the comments from the reviewers and are confident that our response has resulted in substantial improvements to our manuscript.

Editor’s comments:

One of the original reviewers has provided comments on the current manuscript, noting a number of smaller issues that you may choose to correct. Here I note that PLOS ONE does not provide a pre-publication proof so this is the final chance to make these corrections to your paper (e.g the issues with text size in Fig. S1)

**We have made the text size larger for Fig.S1 so it is now legible.**

Please review your reference list to ensure that it is complete and correct. If you have cited papers that have been retracted, please include the rationale for doing so in the manuscript text, or remove these references and replace them with relevant current references. Any changes to the reference list should be mentioned in the rebuttal letter that accompanies your revised manuscript. If you need to cite a retracted article, indicate the article’s retracted status in the References list and also include a citation and full reference for the retraction notice.

**We removed a duplicated reference and have checked that all references are complete and correct.**

Comments Reviewer 2:

Reviewer #2: In the revised version of the manuscript, the Authors have generally addressed most of my concerns, in particular (1) the scientific question has been better defined in the Introduction; (2) the importance of the multivalence interactions has been discussed more profoundly in the last two paragraphs of the Discussion section; (3) the TEM images are now of very good quality, with visible fibril details. Other minor corrections have also been included. Two issues, however, would require some further elaboration.

**We thank the reviewer for these comments and are glad that most of their concerns have been addressed.**

1. As part of the answer to the question of whether the Alexa 488 triplet state can be neglected, the Authors cite their previous publications. I greatly appreciate the Authors' 20 years of experience in the application of FCS. Unfortunately, none of the articles cited in the response directly address this issue: (1) in Biochemistry 2009, the FCS method was not used at all; (2) in JACS 2012, the FCS results were mentioned, but no autocorrelation curves were shown there, so it is difficult to infer the photophysics of Alexa 488 under those conditions; (3) in JBC, the FCS analysis was performed for objects with diffusion times on the order of 1 ms, so possible effects related to the photophysics of the dye in the microsecond range could be neglected. The Authors also prepared an additional Fig. S3. The statistical model comparison shown therein was carried out in the lagtime range above 10 microseconds, i.e. in the range where this effect is no longer visible by its nature; the result showing that the triplet state does not contribute to the autocorrelation curve in the range above 10 us is thus trivial. The analysis in Fig. S3 is inconsistent with Fig. S4, where the whole data in the range from 1 microsecond are analyzed, and the tauD2app results shown in Fig. 3 seem to vary widely, including very small values. If the Authors believe that the photophysics of Alexa 488 does not affect the final results, this assumption should be written explicitely in the Methods, and the analysis in Fig. S3 and S4 should be presented in a consistent manner.
On the other hand, with regard to the possible presence of a free dye in a solution, the Authors have explained that the single-component diffusion model is sufficient.

**We thank the reviewer for pointing out the time scale differences; this was an inadvertent plotting error on our end. We have corrected Fig.S3 to be consistent with Fig.S4. All statistical analysis of fit comparisons was carried out beginning at 1 microsecond and the F-tests still demonstrate that the additional free parameters in the latter two fits are not justified. We have now stated explicitly in the Methods section that we believe the photophysics of Alexa 488 does not affect the final results.**

2. The mean value and standard deviation are applicable only when they refer to multiple repetitions of the same measurement, a Gaussian random variable, i.e., for example, measurements for monomers. However, in a situation where we are studying aggregation and analyzing the relative change in the diffusion time of the resulting complexes (oligomers, aggregates, higher-order structures), the essence of the experiment is that we do not collect multiple repetitions of measurements for the same object, but allow for the heterogeneity of the sample under study. For a heterogeneous sample, the use of the classical statistics is not adequate, but it is more justified to use so-called robust statistics, in which the robust estimators are the median and quartiles. Since the robust statistics should be applied to the aggregates, all data should be analyzed in the same, consistent way. E.g. box plots would be very useful instead of mean and SD, or more sophisticated violin plots, similar to what the Authors did with great success in their work in JBC 2019.

**We thank the reviewer for the suggestion as to how to more effectively represent the FCS data; we have updated the data showing interactions with seeds to include the quartiles (we note these already used the median value as the comparative parameter) in Figure 3, Table 1 and Table S5.**

Moreover, I cannot agree with the Authors’ statement that “some of the reviewer’s concern appears to be misunderstanding. The curves showing the oscillating patterns in S4 Figure are examples of curves that were discarded from our analysis because of our inability to analyze them meaningfully – i.e. the curves that the reviewer points out as problematic were never included in any of the analysis.”, since the curves showing the oscillating patterns are still present in the right-hand column of Fig. S4 (c) and (d) with a legend indicating that these are “the remaining autocorrelation curves following the SSR analysis described in the Materials and Methods”. The diffusion times derived from the autocorrelation curves encompassing the oscillating pattern that reflects the aggregation process occurring during the measurement are determined with much greater uncertainty.

**We agree with the reviewer; while the curves showing the most significant oscillating patterns were eliminated based on our goodness-of-fit (SSR) criteria (this was the basis of our believing there was a misunderstanding by the reviewer), some - including those shown in Fig S4 c and d – were not. To determine whether or not the uncertainty in the τ_D2_ values from curves containing oscillations was generally greater than carried out statistical analysis of the uncertainty in the τ_D2_ values for the curves that do not show oscillations, we normalized the uncertainties calculated by the nonlinear least square fit function in MATLAB to a percentage of the τ_D2_ values for the tau_PRR_ + 60 nM αS seeds dataset. Of the 94 curves included in this dataset post SSR, 11 curves displayed oscillating patterns. Oscillating curves do not demonstrate a larger uncertainty in the fit values as compared to non-oscillating curves: median uncertainty in τ_D2_ for non-oscillating curves = 8.77% (83 curves), and for oscillating curves = 2.69% (11 curves). We attribute this to the large number of data points in each FCS curve; while an oscillation is an obvious deviation from the fit, there are more subtle deviations at other timepoints cumulate to result in comparable uncertainties in the fits.**

Minor points:
1. In the legend to Fig. S4, a typo should be corrected: there is “SRR”, it should be “SSR”.

**We thank the reviewer for catching this typo and have corrected the legend in FigS4.**

2. In the inset of (b) in Fig. S1: the numbers indicating the deconvoluted masses should be larger (now they are illegible).

**We have corrected Fig.S1 to make the numbers larger and legible.**

3. The new figures for the main part of the manuscript now display in darker colors than in the original manuscript, making black lettering on a dark green background (Fig. 1) and points (Fig. 2 and 3) invisible. Also, the gels shown in Fig. 4 are almost black, and thus the results shown on them are invisible. It may be an artifact during the file conversion, but this should be checked.

**We thank the reviewer for pointing this out. The main text figures are all tif files that fall within the standards of the (PACE) digital diagnostic tool,** [**https://pacev2.apexcovantage.com/**](https://urldefense.com/v3/__https:/pacev2.apexcovantage.com/__;!!IBzWLUs!UVNnXw6Tew7W4N0YS2_rGjgggEbvFNF5qfP2hXFH469fxUwRZ7T4XwETg_akSogKL6m_J5Z9n3yaNYflUSJlDDGAVVs$)**, which PLOS uses to ensure that figures meet requirements. When we uploaded them, we checked them using PACE without issue. This is likely is an artifact during conversion; clicking on the link in the figure and downloading results in a figure that is not overly dark. We will work with the journal to make sure the figures are legible.**
